# Systemic Low Dose Corticosteroid Improves Early Postoperative Knee Function and Pain Intensity in Patients Undergoing Unilateral Total Knee Arthroplasty: A Blinded Controlled Randomized Clinical Trial

**DOI:** 10.2106/JBJS.OA.25.00331

**Published:** 2026-03-24

**Authors:** Mohammad H. Ebrahimzadeh, Reza Ganji, Mahdieh Samei, Mohsen Dehghani, Moslem Fallah, Mohammad Mahdi Sarzaeem, Hadi Makhmalbaf, Shayan Zanjanian, Mahla Daliri, Masoumeh Salari, Amir Kachooei

**Affiliations:** 1 Orthopedics Research Center, Ghaem Hospital, Mashhad University of Medical Sciences, Mashhad, Iran; 2 Department of Orthopedic Surgery, School of Medicine, North Khorasan University of Medical Sciences, Bojnurd, Iran; 3 Department of Epidemiology, School of Health, Mashhad University of Medical Sciences, Mashhad, Iran; 4 Department of Orthopedics, Imam Hossein Medical Center, Shahid Beheshti University of Medical Sciences, Tehran, Iran; 5 North Khorasan University of Medical Sciences, Bojnurd, Iran; 6 Rheumatic Diseases Research Center, Mashhad University of Medical Sciences, Mashhad, Iran

## Abstract

**Background:**

This randomized controlled trial evaluates low-dose oral corticosteroids for improving pain and function after total knee arthroplasty (TKA), leveraging their potent anti-inflammatory effects.

**Methods:**

A total of 102 patients who underwent primary unilateral TKA were randomized to receive either 400 mg of celecoxib plus 10 mg of oral prednisolone daily or 400 mg of celecoxib alone. Oral medications started after discharge (24-48 hours after surgery) and continuing for 2 weeks. Follow-up visits were conducted at 1, 2, 4, 12, and 24 weeks postsurgery. The Visual Analog Scale, range of motion (ROM), Knee Society Score, Oxford Knee Score, and sleep quality were evaluated.

**Results:**

Demographic data were similar, except for age, with celecoxib alone group being older (p = 0.005). The celecoxib + prednisolone group experienced significantly lower Visual Analog Scale pain scores at 1 week (mean difference [MD]: −0.81; 95% confidence interval [CI]: −1.59 to −0.03) and 2 weeks (MD: −0.99; 95% CI: −1.87 to −0.09) postoperatively. Age-adjusted results confirmed the reduction in pain with a slight difference, although it was not statistically significant. Knee function scores showed statistically significant improvement in the celecoxib + prednisolone group at 2 weeks (MD: 12.96; 95% CI: 0.21-26.13). Improvement in knee function scores was reduced by about half in age-adjusted analysis and was not statistically significant (MD: 6.25, p = 0.35). ROM demonstrated significant difference at 4 weeks (MD: 6.66; 95% CI: 0.44-12.86). Sleep quality showed significant improvement in the celecoxib + prednisolone group at 2 weeks (MD: 0.84; 95% CI: 0.05-1.64). Improvement was clinically confirmed by controlling for the effect of age with borderline significance. Uncertainty was observed due to wide CIs in some results.

**Conclusion:**

Oral low-dose prednisolone administered early after TKA resulted in significant reductions in pain and improvements in sleep quality, ROM, and Knee Society Score during the early postoperative period. However, these improvements did not consistently reach the minimal clinically important difference. Adjusted analyses accounting for age suggested modest benefits, but wide CIs and small effect sizes warrant cautious interpretation. To optimize dosing regimens and assess the long-term effects of oral corticosteroids in TKA recovery protocols, large-scale randomized controlled trials are necessary.

**Level of Evidence:**

Therapeutic Level I. See Instructions for Authors for a complete description of levels of evidence.

## Introduction

Total knee arthroplasty (TKA) is the gold standard for treating end-stage knee osteoarthritis (OA), significantly improving quality of life and pain relief^1-4^. Effective early postoperative pain management is crucial, as it reduces hospital stays, improves patient satisfaction, and fosters better rehabilitation outcomes^3,5,6^. Many patients also experience chronic pain and functional impairment preoperatively, heightening expectations for rapid recovery.

Glucocorticoids, known for their potent anti-inflammatory and antiemetic properties, are widely used in perioperative TKA pain management^7-10^. Agents, such as dexamethasone, prednisolone, and triamcinolone, administered intravenously or periarticularly^3^, reduce postoperative pain, swelling, opioid use, and nausea, aiding early recovery^11^.

This study proposes that low-dose oral corticosteroids may work together to alleviate postoperative pain, reduce inflammation, and promote early functional recovery. In contrast to earlier research with shorter follow-up durations, our study examines outcomes up to 6 months postsurgery and includes a wider array of clinical measures, offering a more thorough understanding of the prolonged advantages of oral prednisolone following TKA.

## Materials and Methods

### Study Setting

This blinded 2-arm parallel randomized controlled trial (RCT) was conducted in 3 tertiary hospitals from April to September 2024. Informed consent was obtained. The study followed CONSORT guidelines^12^, received regional branch of the national ethics committee approval (IR.MUMS.IRH.REC.1402.200), and was registered with the Iranian Registry of Clinical Trials (IRCT20221228056952N2).

### Participants

Eligible participants were adults 50 years or older scheduled for primary unilateral TKA due to advanced OA, following the American Association of Hip and Knee Surgeons surgical criteria. Exclusion criteria included history of alcohol or drug abuse, pregnancy or breastfeeding, chronic glucocorticoid use, therapeutic anticoagulation, corticosteroid hypersensitivity, active systemic infection, or immunocompromised. Patients with rheumatoid arthritis, metastatic disease, chronic kidney disease (glomerular filtration rate <60 mL/min/1.73 m), unstable cardiovascular disease, diabetic neuropathy, major neurological disorders, or psychiatric illnesses were also excluded.

### Sample Size Determination

The sample size was calculated based on mean Visual Analog Scale (VAS) pain scores from Tammachote et al., with dexamethasone and control groups showing means of 36 ± 23 and 51 ± 28 at 21 hours postoperatively, respectively. Assuming a 95% confidence level (α = 0.05) and 80% power (β = 0.20)^13^, 46 patients per group were needed (total 92). Accounting for a 10% dropout rate, the sample size was increased to 102. Thus, 102 eligible patients were randomized to study groups with 51 patients in each group.

### Interventions

Basic patient data, including age, sex, and medical history, were recorded using a standardized form. Eligible patients were randomized by blocked randomization into 2 groups. Both groups received standard analgesia, including intraoperative cocktail injections of 100 to 150 mg bupivacaine, 0.5 mg epinephrine, and 30 mg/1 mL ketorolac in 100 mL saline. The celecoxib + prednisolone group received a daily dose of 400 mg celecoxib (200 mg tablets twice daily) plus 10 mg oral prednisolone (two 5 mg tablets) for 2 weeks. By contrast, during the same period, celecoxib alone group received 400 mg of celecoxib alone daily (two 200 mg tablets). Postoperative anticoagulation and rehabilitation protocols were the same for both groups. A standardized tranexamic acid protocol was applied in all patients, consisting of 1 gram administered intravenously before surgical incision, followed by 1 gram injected intra-articularly immediately after wound closure.

### Surgical Procedure

All patients underwent TKA under general anesthesia without a tourniquet, using a midline incision and anteromedial arthrotomy through the midvastus approach. Mechanical alignment was applied, and a Persona Zimmer Biomet prosthesis was used. Patellar resurfacing was not performed, and no Hemovac drains were placed. Patients commenced ambulation with a walker on the first postoperative day and were discharged 24 to 48 hours after surgery.

### Study Outcomes

The primary outcome was postoperative pain intensity measured using VAS. Secondary outcomes included functional assessments using the Persian version of the Knee Society Score (KSS)^14^, Oxford Knee Score (OKS)^15^, and objective measurement of knee range of motion (ROM). All measurements were performed by a single trained clinician using a standardized protocol, averaging 2 readings per assessment to reduce variability. To assess intraobserver error, 2 independent measurements were taken for each assessment by the same clinician. The reproducibility was evaluated using the intraclass correlation coefficient, which demonstrated relatively high agreement (intraclass correlation coefficient = 0.91, 95% confidence interval [CI]: 0.88-0.97) between 2 readings.

In addition, complications and sleep quality were evaluated as important patient-centered outcomes. Sleep quality was assessed by an 11-point Numeric Rating Scale (0 = poor to 10 = excellent)^16^. Assessments were conducted preoperatively and at 1, 2, 4, 12, and 24 weeks postoperatively. Demographic variables, including age, BMI, sex, occupation, and education, were recorded. All evaluations were performed by blinded personnel independent of the surgical team to reduce bias.

### Randomization

A computer-generated sequence with blocked randomization was used to ensure balanced groups in this RCT. A total of 24 blocks of 4 and 2 blocks of 3 were generated. Following eligibility screening and informed consent acquisition, participants were allocated to the celecoxib + prednisolone or celecoxib alone group in a 1:1 ratio according to the randomized block sequence. The randomization process was concealed. Allocation concealment was rigorously preserved by using 102 sealed, opaque envelopes, which were exclusively managed by an independent secretary with no involvement in outcome assessment or data recording.

### Blinding

Surgeons, trained clinician, and the outcome assessor were blinded to the treatment allocation. All preoperative and postoperative data were collected by a team independent of the surgeons to ensure unbiased assessment of the study outcomes.

### Statistical Analysis

Data were analyzed using SPSS version 22.0 (SPSS) software. Descriptive statistics, including mean, SD, and percentage, were reported according to the variable scales. Normality was assessed using the Kolmogorov-Smirnov test and visually through histograms. Comparisons between groups were performed using χ^2^ or Fisher exact tests for qualitative variables and independent samples *t* test or Mann-Whitney U test for quantitative variables, as appropriate. To control the effect of age difference at baseline, Analysis of Covariance (ANCOVA) or nonparametric ANCOVA using the Quade test was used. Repeated measures ANCOVA evaluated changes over time across multiple postoperative follow-ups. Statistical significance was set at p < 0.05.

## Results

### Descriptive Results

**Fig. 1:**
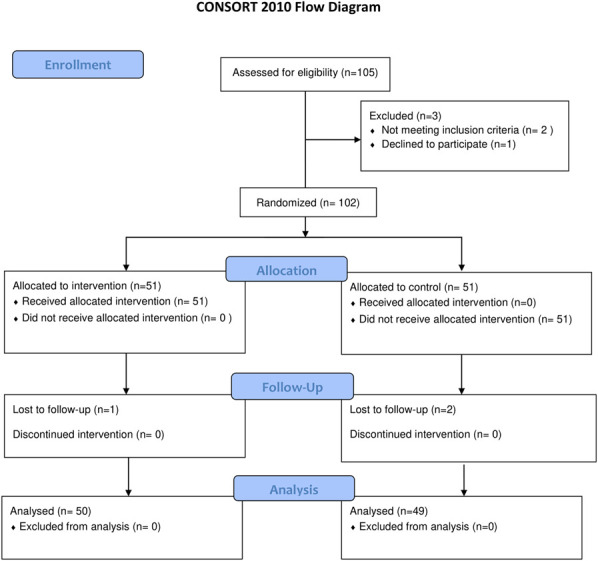
CONSORT flow diagram of the protocol of the study.

Of 102 patients enrolled, 3 were lost to follow-up, leaving 50 in the celecoxib + prednisolone group and 49 in the celecoxib alone group for analysis. Baseline characteristics were similar between groups, with no significant differences in sex, education, occupation, operated knee side, BMI, or comorbidities (p > 0.05). Common comorbidities in the celecoxib + prednisolone group included hypertension (32%), cardiovascular disease (6%), and renal disease (4%). In the celecoxib alone group, hypertension (37%) and cardiovascular disease (6%) were most frequent. Our calculated mean Charlson Comorbidity Index (CCI) scores for the celecoxib + prednisolone group (0.14) and the celecoxib alone group (0.06) are consistent with reported ranges in similar clinical studies, supporting the comparability and validity of this index for our study population. Thus, the reported CCI values effectively characterize the relative comorbidity burden between treatment groups (Table I and Fig. 1).

**TABLE I:** Baseline Characteristics of the Participants

	Celecoxib + Prednisolone (n = 51)		Celecoxib alone (n = 51)		
Variables	No.	%	No.	%	p
Sex					0.33^a^
Male	13	25.5	9	17.6	
Female	38	74.5	42	82.4	
Total	51	50	51	50	
Education					0.36^a^
Illiterate	25	49.0	28	54.9	
Under diploma	5	9.8	7	13.7	
Diploma	13	25.5	12	23.5	
Bachelor	4	78	14	7.8	
Unknown	4	7.8	0	0	
Occupation					0.72^a^
Self-employment	1	2.0	2	3.9	
Worker	2	3.9	1	2.0	
Farmer	1	2.0	2	3.9	
Retired	2	3.9	5	9.8	
Housekeeper	39	76.5	38	74.5	
Unknown	6	11.8	3	5.9	
Side					1.00^a^
Right	20	39.2	20	39.2	
Left	30	58.8	30	58.8	
Unknown	1	2.0	1	2.0	
Underlying disease					1.00^a^
Yes	22	43.1	21	41.1	
No	28	54.9	28	55.0	
Unknown	1	2.0	2	3.9	
Age					
Year, Mean ± SD	64.98 ± 8.56		69.42 ± 6.58		0.005^b,*^
BMI					0.91^c^
Mean ± SD	31.11 ± 9.59		30.88 ± 9.96		

BMI = body mass index.

a χ^2^.

b *t* test.

c Mann-Whitney.

* Significant at 0.05 level.

### VAS Pain Score

The celecoxib + prednisolone group reported significantly lower pain at rest early after surgery (6.5 ± 2.19) vs. celecoxib alone group (7.30 ± 1.69) at week 1 (MD: −0.81; p = 0.04) and similarly at week 2 (MD: −0.99; p = 0.03). No significant differences were found for pain during movement. Both groups improved gradually over 24 weeks (Table II and Supplementary Fig. 1a and b). Age-adjusted results confirmed the reduction in pain with a slight difference, although it was not statistically significant (MD: −0.57; p = 0.15 at week 1 and MD: −0.71; p = 0.12 at week 2).

**TABLE II:** Comparison of Postoperative Sleep Quality and Pain Scores at Rest and During Movement Between Groups Using the Visual Analog Scale

		Celecoxib + Prednisolone (n = 50)	Celecoxib Alone (n = 49)				
Clinical Outcomes	Follow-up Time	(Mean ± SD)	(Mean ± SD)	Mean Difference^a^ (95% CI)	p	Age-Adjusted Mean Difference (95% CI)	p
	Preop	4.64 ± 2.64	4.84 ± 2.39	−0.20 (−1.20 to 0.79)	0.68^b^	−0.43 (−1.48 to 0.60)	0.40^c^
	Postop week 1	6.49 ± 2.19	7.30 ± 1.69	−0.81 (−1.59 to −0.03)	0.04^b,*^	−0.57 (−1.38 to 0.23)	0.15^c^
VAS at rest	Postop week 2	5.71 ± 2.55	6.70 ± 1.85	−0.99 (−1.87 to −0.09)	0.03^b,*^	−0.71 (−1.63 to 0.21)	0.12^c^
	Postop week 4	3.74 ± 2.21	4.08 ± 2.32	−0.34 (−1.26 to 0.58)	0.46^b^	−0.15 (−0.99 to 0.90)	0.92^c^
	Postop week 12	2.20 ± 2.54	2.32 ± 2.06	−0.12 (−1.04 to 0.80)	0.19^d^	−0.03 (−1.11 to 0.95)	0.65^e^
	Postop week 24	1.49 ± 2.06	1.64 ± 2.25	−0.15 (−1.01 to 0.71)	0.92^d^	−0.30 (−1.21 to 0.61)	0.51^e^
	Preop	8.34 ± 1.23	7.96 ± 1.63	0.38 (−0.19 to 0.95)	0.19^b^	0.26 (−0.34 to 0.87)	0.39^c^
	Postop week 1	7.14 ± 2.15	7.68 ± 1.43	−0.54 (−1.26 to 0.19)	0.14^b^	−0.38 (−1.15 to 0.37)	0.31^c^
VAS during activity	Postop week 2	6.59 ± 2.66	7.06 ± 1.88	−0.47 (−1.38 to 0.45)	0.31^b^	−0.25 (−1.22 to 0.71)	0.60^c^
	Postop week 4	5.13 ± 2.36	4.80 ± 2.33	0.33 (−0.62 to 1.28)	0.49^b^	0.67 (−0.30 to 1.65)	0.17^c^
	Postop week 12	3.57 ± 2.55	2.92 ± 2.26	0.65 (−0.31 to 1.61)	0.19^d^	0.76 (−0.26 to 1.78)	0.14^e^
	Postop week 24	1.84 ± 2.20	1.39 ± 1.85	0.45 (−0.36 to 1.26)	0.40^d^	0.41 (−0.45 to 1.28)	0.34^e^
	Postop week 1	7	7	0	1.00	0	1.00
	Postop week 2	7.33 ± 1.83	6.48 ± 2.14	0.84 (0.05-1.64)	0.03^b,*^	0.73 (−0.09 to 1.56)	0.08^c^
Sleep quality	Postop week 4	8 ± 1.64	7.38 ± 2.12	0.62 (−0.15 to 1.39)	0.11^b^	0.52 (−0.29 to 1.33)	0.20^c^
	Postop week 12	8.63 ± 1.82	8.72 ± 1.50	−0.08 (−0.75 to 0.57)	0.88^d^	0.10 (−0.56 to 0.79)	0.77^e^
	Postop week 24	8.98 ± 1.46	8.94 ± 1.87	0.04 (−0.63 to 0.71)	0.54^d^	0.17 (−0.53 to 0.88)	0.62^e^

VAS = Visual Analog Scale.

a Differences were calculated using celecoxib alone group as baseline; a negative value indicates a lower score in the celecoxib + prednisolone group.

b Independent samples *t* test.

c ANCOVA.

d Mann-Whitney.

e Nonparametric ANCOVA.

* Significant at 0.05 level.

### Sleep Quality

Postoperative sleep quality was similar between groups except at week 2, when the celecoxib + prednisolone group reported significantly better sleep (7.33 ± 1.83 vs. 6.48 ± 2.14; p = 0.03). Age-adjusted analysis showed borderline significance (MD: 0.73; p = 0.08). Both groups showed substantial improvements in mobility and pain relief after surgery (Table II and Supplementary Fig. 1c).

### Oxford Knee Score

Both groups showed improved knee function and mobility postsurgery, indicated by increasing OKS. Preoperatively, OKS was slightly lower in the celecoxib + prednisolone group but not significantly different. During follow-up, the celecoxib + prednisolone group maintained numerically higher OKS values at weeks 1, 2, and 4. At week 24, the celecoxib + prednisolone group scored 51.4 ± 23.34 vs. 46.6 ± 18.37 in the celecoxib alone group (MD: +4.81; p = 0.25), clinically important but not statistically significant (Table III and Supplementary Fig. 2a). Age-adjusted results confirmed the improvement in OKS, with a slight difference, although it was not statistically significant (MD: 4.34; p = 0.33).

**TABLE III:** Comparison of Oxford Knee Score, Knee Society Score, Knee Function Scores, and Range of Motion Questionnaire Scores Between Groups During Follow-up Times

		Celecoxib + Prednisolone (n = 50)	Celecoxib Alone (n = 49)				
Clinical Outcomes	Follow-up Time	(Mean ± SD)	(Mean ± SD)	Mean Difference^a^ (95% CI)	p	Age-Adjusted Mean Difference (95% CI)	p
OKS	Preop	15.46 ± 6.99	16.90 ± 7.81	−1.44 (−4.39 to 1.51)	0.33^b^	−1.21 (−4.35 to 1.93)	0.44^c^
	Postop week 1	20 ± 9.85	17.71 ± 9.31	2.29 (−1.55 to 6.13)	0.24^b^	0.50 (−3.41 to 4.41)	0.80^c^
	Postop week 2	23.94 ± 9.69	21.55 ± 8.70	2.39 (−1.30 to 6.08)	0.20^b^	1.07 (−2.76 to 4.90)	0.58^c^
	Postop week 4	31.83 ± 9.54	29.49 ± 8.28	2.33 (−1.29 to 5.97)	0.20^b^	1.05 (−2.68 to 4.79)	0.57^c^
	Postop week 12	35.49 ± 8.76	36.78 ± 8.64	−1.29 (−4.76 to 2.18)	0.46^b^	−1.40 (−5.10 to 2.28)	0.45^c^
	Postop week 24	51.37 ± 23.34	46.56 ± 18.37	4.81 (−3.56 to 13.17)	0.25^b^	4.34 (−4.56 to 13.25)	0.33^c^
KSS	Preop	44.06 ± 32.88	45.31 ± 29.56	−1.25 (−13.92 to 11.42)	0.84^b^	−1.60 (−14.91 to 11.71)	0.81^c^
	Postop week 1	43.58 ± 17.22	44.77 ± 14.27	−1.19 (−7.59 to 5.22)	0.71^b^	−1.17 (−7.96 to 5.63)	0.73^c^
	Postop week 2	49.29 ± 15.75	47.45 ± 14.93	1.84 (−4.32 to 7.99)	0.55^b^	0.59 (−6.95 to 5.76)	0.85^b^
	Postop week 4	62.59 ± 14.38	60.19 ± 16.71	2.41 (−4.03 to 8.82)	0.46^b^	0.05 (−6.54 to 6.65)	0.98^c^
	Postop week 12	72.27 ± 14.81	74.64 ± 15.87	−2.37 (−8.05 to 3.75)	0.44^b^	−2.20 (−8.70 to 4.30)	0.50^c^
	Postop week 24	74.92 ± 13.92	80.64 ± 19.57	−5.72 (−12.51 to −1.06)	0.02^d,*^	−4.25 (−11.41 to 2.91)	0.24^e^
KFS	Preop	54.90 ± 28.60	58.80 ± 23.35	−3.90 (−14.30 to 6.50)	0.45^b^	−4.11 (−15.07 to 6.83)	0.45^c^
	Postop week 1	24.49 ± 30	17.40 ± 29.49	7.09 (−4.90 to 19.09)	0.29^d^	5.27 (−10.03 to 14.58)	0.71^e^
	Postop week 2	44.39 ± 35.62	31.43 ± 29.82	12.96 (0.21-26.13)	0.05^b,**^	6.25 (−7.02 to 19.54)	0.35^c^
	Postop week 4	60.33 ± 25	60.83 ± 20.96	−0.50 (−9.94 to 8.92)	0.91^b^	−3.04 (−12.89 to 6.81)	0.54^c^
	Postop week 12	79.59 ± 18.33	83.47 ± 18.29	−3.88 (−11.22 to 3.46)	0.22^d^	−4.53 (−12.35 to 3.27)	0.25^e^
	Postop week 24	87.65 ± 14.18	90.08 ± 14.35	−2.43 (−8.12 to 3.26)	0.18^d^	−2.56 (−8.59 to 3.47)	0.40^e^
ROM	Preop	101.76 ± 22.17	98.67 ± 27.70	3.09 (−6.84 to 13.03)	0.53^b^	3.16 (−7.47 to 13.79)	0.55^c^
	Postop week 1	86.60 ± 17.18	82.76 ± 20.61	3.84 (−3.72 to 11.41)	0.31^b^	2.21 (−5.76 to 10.20)	0.58^c^
	Postop week 2	91.38 ± 16.27	87.76 ± 16.55	3.62 (−2.92 to 10.17)	0.16^d^	4.01 (−2.26 to 10.30)	0.20^e^
	Postop week 4	104.10 ± 14.05	97.44 ± 16.43	6.66 (0.44-12.86)	0.05^d,**^	4.53 (−1.90 to 10.98)	0.16^e^
	Postop week 12	111.90 ± 14.35	109.49 ± 16.62	2.41 (−3.78 to 8.60)	0.44^b^	1.19 (−5.59 to 7.44)	0.77^c^
	Postop week 24	118.80 ± 11.40	116.94 ± 12.06	1.86 (−2.82 to 6.54)	0.43^b^	1.28 (−3.69 to 6.25)	0.61^c^

KSS = Knee Society Score, KFS = Knee Function Score, OKS = Oxford Knee Score, Pre-op = preoperative, Postop = postoperative, and ROM = range of motion.

a Differences were calculated using celecoxib alone group as baseline; a negative value indicates a lower score in the celecoxib + prednisolone group.

b Independent samples *t* test.

c ANCOVA.

d Mann-Whitney.

e Nonparametric ANCOVA.

* Significant at 0.05 level.

** Borderline significant.

### Knee Society Score

The KSS improved steadily across both groups. No significant differences were observed early postoperatively, but at week 24, the celecoxib alone group had significantly higher functional scores (80.64 ± 19.57 vs. 74.92 ± 13.92; p = 0.02), indicating better long-term knee function without prednisolone. Age-adjusted results confirmed the reduction in KSS with a slight difference; however, it was not statistically significant (MD: −4.25; p = 0.24). At week 2, the celecoxib + prednisolone group had borderline improved Knee function scores (KFS) (44.39 ± 35.62 vs. 31.43 ± 29.82; p = 0.05), suggesting a temporary functional benefit that diminished over time. Improvement in KFS was reduced by about half in age-adjusted analysis and was not statistically significant (MD: 6.25; p = 0.35) (Table III, Supplementary Fig. 2b, and 2c).

### Range of Motion

Active ROM improved postoperatively in both groups with no significant differences at most time points. At week 4, the celecoxib + prednisolone group showed borderline significantly greater ROM (104.10 ± 14.05 vs. 97.44 ± 16.43; p = 0.05). Age-adjusted results confirmed the improvement in ROM with a slight difference; however, it was not statistically significant (MD: 4.53; p = 0.16) (Table III and Supplementary Fig. 2d).

### Complications

No cases of superficial wound dehiscence, surgical site infection, deep vein thrombosis, or thromboembolic events were observed in either group during follow-ups.

## Discussion

This clinical trial found that adding low-dose oral prednisolone (10 mg daily for 2 weeks) to a multimodal pain management regimen after TKA significantly reduces early postoperative pain and improves functional outcomes such as ROM and KSS. The celecoxib alone group was significantly older (69 ± 6.6 years) than the celecoxib + prednisolone group (65 ± 8.6 years; p = 0.005), but this difference is attributed to chance given the randomized design.

Patients who received prednisolone reported significantly greater pain relief at rest, which may be explained by variations in patient demographics and the timing of follow-up assessments^17^. In addition, these patients exhibited an average increase of 6.66° in ROM at 4 weeks after surgery, reflecting improved early mobility. This level of improvement is comparable to or exceeds values documented in previous studies, thereby reinforcing the efficacy of our intervention^17^. These early benefits align with corticosteroids’ anti-inflammatory effects that reduce surgical inflammation.

Recent studies suggest that the minimal clinically important difference (MCID) for the VAS is about 1.65 to 2.0 on a scale of 0 to 10^18^. The observed 4.81-point improvement in OKS falls just below the accepted 5.0-point MCID threshold for TKA^19^. Thus, no clinically significant difference was demonstrated between groups.

The study found prednisolone improved sleep quality significantly at 2 weeks postoperatively. Despite the lack of previous research specifically examining prednisolone’s effect on sleep after TKA, reduced pain likely contributed to better sleep, as pain disrupts sleep architecture. Improved sleep may promote overall recovery and well-being^20,21^.

Despite its strengths, the study has limitations. There is a growing body of research supporting the use of oral corticosteroids as an effective strategy for early postoperative management following total joint replacement^22^. To the best of our knowledge, few randomized trials have specifically studied the effects of oral prednisolone on short-term TKA outcomes such as OKS, KSS, and ROM. The focus was on clinical outcomes, without serum inflammatory markers, which could elucidate prednisolone’s biological effects. In addition, the benefits of prednisolone appeared to diminish during the short-term postoperative period, with no significant differences in pain scores beyond this time frame. This raises questions about its role in preventing chronic pain development after TKA^22^.

In our clinical setting, narcotics are administered only during inpatient hospitalization, typically within 24 to 48 hours after surgery. Owing to regional regulations, oral narcotics are not available for outpatient prescription. Consequently, no patient received narcotics after leaving the hospital. On discharge, all patients followed a standardized non-narcotic regimen of non-steroidal anti-inflammatory drugs and acetaminophen. Randomization and trial medication (prednisolone) initiation occurred at discharge (24-48 hours after surgery). The primary outcome was pain intensity at 1 week postsurgery, a time point when inpatient narcotic effects would have long dissipated, and all patients were on a uniform, non-narcotic analgesic protocol. Although quantifying inpatient narcotic use is a limitation, it was not a confounding variable for our primary outcome. Our study was intentionally designed to assess the effect of adding prednisolone to a standard, non-narcotic, postdischarge regimen.

Prednisolone’s analgesic effects stem from inhibiting inflammatory mediators and reducing nociceptive input, which decreases pain, joint stiffness, and anxiety, facilitating better rehabilitation. However, ROM and functional recovery are influenced by many factors beyond medication, including surgical technique, implant design, preoperative conditioning, and rehabilitation quality^7^.

Several studies have examined the efficacy and safety of systemic steroids in total knee and hip arthroplasty. Most report benefits such as reduced postoperative pain, opioid consumption, and shorter hospital stay. However, findings on functional improvements and adverse effects such as infection and hyperglycemia remain inconsistent across studies. This systematic review synthesizes these findings to provide an overview of current evidence and identify gaps for future research^11,23^.

Further large, well-powered randomized trials with longer follow-up and biomarker analyses are needed to clarify oral prednisolone's role in optimizing outcomes after TKA.

In conclusion, administration of oral prednisolone led to a significant reduction in pain and statistically significant improvement in sleep quality, ROM, and KSS in the early postoperative period. However, these findings did not reach the level of minimal clinically important difference. Findings warrant cautious interpretation due to wide CIs and potential bias from a small sample size. Large-scale RCTs with stratified analyses are needed to confirm these results and clarify long-term benefits and risks of oral prednisolone in TKA recovery.

## Appendix

Supporting material provided by the authors is posted with the online version of this article as a data supplement at jbjs.org (http://links.lww.com/JBJSOA/B118). This content was not copyedited or verified by JBJS.
